# Overcoming Antibiotic Resistance: Playing the ‘Silver Nanobullet’ Card

**DOI:** 10.3390/ma15030932

**Published:** 2022-01-26

**Authors:** Morena Nocchetti, Elisa Boccalon, Monica Pica, Nicoletta Maria Rosaria Giordano, Francesco Finori, Donatella Pietrella, Antonio Cipiciani

**Affiliations:** 1 Department of Pharmaceutical Sciences, University of Perugia, Via del Liceo, 1, 06123 Perugia, Italy; monica.pica@unipg.it (M.P.); nicoletta.giordano@hotmail.it (N.M.R.G.); 2 Department of Industrial Engineering, University of Salerno, Via Giovanni Paolo II, 132, 84084 Fisciano, Salerno, Italy; elisa.boccalon@gmail.com; 3 Department of Chemistry, Biology and Biotechnology, University of Perugia, Via Elce di Sotto, 8, 06123 Perugia, Italy; cescofinori@hotmail.it (F.F.); antonio.cipiciani@libero.it (A.C.); 4 Microbiology and Clinical Microbiology, Department of Medicine and Surgery, University of Perugia, Piazzale Gambuli, 1, 06129 Perugia, Italy; donatella.pietrella@unipg.it

**Keywords:** layered double hydroxides, hydrotalcites, antibiotic resistance, silver, antibacterial activity

## Abstract

Enhancing the antibacterial activity of old antibiotics by a multitarget approach, such as combining antibiotics with metal nanoparticles, is a valuable strategy to overcome antibacterial resistance. In this work, the synergistic antimicrobial effect of silver nanoparticles and antibiotics, immobilized on a solid support, was investigated. Nanometric layered double hydroxides (LDH) based on Zn(II) and Al(III) were prepared by the double microemulsion technique. The dual function of LDH as an anionic exchanger and support for metal nanoparticles was exploited to immobilize both silver and antibiotics. Cefazolin (CFZ), a β-lactam, and nalidixic acid (NAL), a quinolone, were selected and intercalated into LDH obtaining ZnAl-CFZ and ZnAl-NAL samples. These samples were used for the growth of silver nanoparticles with dimension ranging from 2.5 to 8 nm. Silver and antibiotics release profiles, from LDH loaded with antibiotics and Ag/antibiotics, were evaluated in two different media: water and phosphate buffer. Interestingly, the release profiles are affected by both the acceptor media and the presence of silver. The synergistic antibacterial activity of LDH containing both silver and antibiotics were investigated on gram-positives (*Staphylococcus aureus* and *Streptococcus pneumoniae*) and gram-negatives (*Pseudomonas aeruginosa*) and compared with the plain antimicrobials and LDH containing only antibiotics or silver.

## 1. Introduction

Shortly after they were discovered, it was clear that antibiotics would have become a mixed blessing. Soon, the alarm was raised about their undiscriminating use and irresponsible abuse [1], which contributed to jeopardizing their efficacy through the development of drug resistance mechanisms. As a consequence, the initial optimism surrounding this fortuitous discovery was replaced by concerns about the risk that the hailed ‘miracle drug’ represented if thoughtlessly assumed [2]. Now more than ever, the escalating crisis of antibiotic resistance is pressing science to take action in order to stave off a tremendous decline towards a post-antibiotic era [3]. This quest includes the diffusion of new drugs [4,5], the repurpose of older ones [6], the administration of mixed antibiotic regimens [7,8], and the testing of target-based approaches focused on the development of specific inhibitors [9,10]. Another strategy involves the combination of antibiotics with metal nanoparticles (NPs). Coupling traditional antibiotics with nanotechnology can circumvent drug resistance by furnishing alternative routes to induce bacterial death or inhibition (e.g., direct interaction with cell wall components [11], cleaving to DNA and RNA [12], generation of reactive oxygen species [13], disruption of electron transport chain [14], damage of efflux pumps [15], etc.), which can work synergistically with that of standard drugs [16], preserving or even enhancing their effectiveness. Thanks to their broad spectrum of activity, silver NPs are considered to be among the most promising antibacterial nanomedical systems, and the benefits of their conjunctive action with several antibiotics have been reported on many occasions [17,18]; however, depending on the nature of the antibiotic tested, antagonistic responses have been observed as well [19]. Different factors have been adduced to explain this discrepancy, involving not only specific drug-silver interactions but also the size and shape of the nanoparticles [20,21]. Finally, some authors have pointed out that a significant influence can be played by the stabilizing agents used for the NPs synthesis [22]. Capping agents are known to control the steric stabilization of the particles, prevent their aggregation, and improve the bioavailability of the drug [22,23]; therefore, the choice of the most suitable stabilizing agent can become crucial and seriously affect the final results. To work around this problem, silver NPs could be synthesized without any surfactant and can be anchored directly over a support. On this regard, thanks to their biocompatibility [24,25], compositional versatility, and easy functionalization, layered double hydroxides (LDHs) constitute an ideal substrate for the deposition of nanocrystals [26,27]. LDH are characterized by the general formula [M(II)_1−x_M(III)_x_(OH)_2_](A^−n^)_x/n_·mH_2_O, where M(II) can be Mg, Zn, Cu, etc.; M(III) is mainly Al, Ga, or Fe; A^−n^ are the anions, located in the interlayer region, that balance the positive charges of the layers [28]. LDH with antimicrobial properties can be prepared by selecting a proper composition of the layers and of the interlayer and by exploiting the surface properties. The right choice of intralayer metals with antimicrobial activity such as Cu, Zn, or Ga [29,30], or the intercalation of antibiotics, allowed LDH with good antibacterial properties to be obtained [31,32]. Immobilization of metal NPs, such as AgNPs, was achieved thanks to the presence of layer surface OH groups, acting as stabilizing agents. Moreover, the reaction between the charge balancing anions of the LDH and silver nitrate resulted in the formation of insoluble negatively charged salts of silver, stabilized by interaction with the positive charge of the layers [26,27]. In addition to these features, an attractive aspect of the LDH is the possibility to tune the crystal size. In this connection, LDH with nanometric size can be obtained by the double microemulsion technique [33]; the obtained particles maintain the reactivity of micrometric ones and can be considered good candidates as drug carriers in the field of nanomedicine [34,35].

In this work, the ion exchange properties of nanometric LDH were combined with the ability to immobilize silver nanoparticles to obtain LDHs with dual function: drug releaser and nanoparticle host. The first part of the work was devoted to verify the interactions of Ag NPs with selected antibiotics intercalated into nanometric LDHs, over which the Ag NPs are attached. Specifically, two antibacterial agents were chosen: cefazolin (CFZ) belonging to the class of β-lactamases and nalidixic acid (NAL), a quinolone. The selected antibacterial agents possess a carboxylic function that, properly deprotonated, makes these anionic species suitable to be intercalated into LDH. Before evaluating their synergistic behavior with silver, the release profiles of each LDH-drug system were studied in two different media, water and phosphate buffer (PBS). Afterwards, Ag NPs were deposited on the LDH surfaces, and the antibacterial activity was investigated by performing MIC assays on two gram-positives (*Staphylococcus aureus* and *Streptococcus pneumoniae*) and a gram-negative (*Pseudomonas aeruginosa*). The data collected proved that Ag NPs are able to assist the antimicrobial activity of antibiotics, enhancing it with remarkable results especially against gram-negative bacteria.

## 2. Materials and Methods

### 2.1. Materials

Zn(NO_3_)_2_·6H_2_O and Al(NO_3_)_3_·9H_2_O were purchased by Riedel-de-Haën (Selze, Germany). AgNO_3_ by Carlo Erba (Milan, Italy), cetyltrimethylammonium bromide (CTABr), n-butanol, isooctane, NH_3_, cefazolin, and nalidixic acid are a Sigma-Aldrich (Darmstadt, Germany) product. Deionized water was obtained by a reverse osmosis process using a Milli Q system (Millipore, Rome, Italy).

### 2.2. Synthesis of ZnAl-Br LDH

LDH was synthesized according to the double microemulsion technique [33]. Two microemulsions, designated A and B, were prepared by dispersing 12.5 g (0.034 mol) of a cationic surfactant, cetyltrimethylammonium bromide (CTABr), in 15.6 mL (0.17 mol) of n-butanol, 36.2 mL (0.22 mol) of isooctane, and 13.5 mL of aqueous phase. The aqueous phase of A is composed of Zn(NO_3_)_2_·6H_2_O 0.4 M and Al(NO_3_)_3_·9H_2_O 0.125 M, while the aqueous phase of B consists of a 2.5 M NH_3_ solution. The two microemulsions, A and B, were mixed to obtain the precipitation of ZnAl LDH in the reverse micelles. The resulting system was stirred at room temperature for 15 min, then aged at 80 °C for 15 h. After aging, the particles were recovered by centrifugation (12,000 rpm, 10 min), and a semi-transparent gel was obtained. The gel was washed three times with 30 mL of methanol/chloroform mixture (1:1 *v*/*v*), three times with 30 mL of CO_2_-free de-ionized water, and again once with 30 mL of a methanol/chloroform mixture (1:1 *v*/*v*) and then dried at room temperature under vacuum. The Zn, Al, and bromide content in the sample was determined by inductively coupled plasma and ionic chromatography analysis, respectively; the LDH composition is [Zn_0.72_Al_0.28_(OH)_2_]Br_0.28_·0.7H_2_O (hereafter ZnAl-Br) (ion exchange capacity, IEC = 2.27 mmol/g).

### 2.3. Preparation of Acetate LDH

In order to facilitate the diffusion of antibacterial species within the interlayer space of LDH, the interlayer distance of LDH was increased by replacing bromide ions with acetate through an ion exchange procedure. Specifically, 250 mg of ZnAl-Br were added to 25 mL of a 2.5 M CH_3_COONa solution (IEC/CH_3_COO^−^ molar ratio = 9). The mixture was stirred under mild agitation for 96 h at room temperature, then centrifuged (12,000 rpm, 10 min) and washed with decarbonated water (1 × 30 mL). The product was used in the wet form and named as ZnAl-Ac.

### 2.4. Intercalation of Antibiotics into LDH

An amount of 0.1 M solutions of each antibiotic were prepared by dissolving them in decarbonated water. Cefazolin (CFZ) was used as sodium salt (50 mg CFZ/mL). Instead, to ensure deprotonation of the carboxylic group of nalidixic acid (NAL), about 2 mL of 1 M NaOH was dropped in the solution of nalidixic acid, prepared dissolving 450 mg of NAL in 16 mL of decarbonated water, up to pH 10 (25 mg NAL/mL). The wet ZnAl-Ac, prepared as reported in the paragraph 2.3 and corresponding to 250 mg of ZnAl-Br (containing 0.57 mmol of exchangeable CH_3_COO^−^), was mixed with 11.4 mL of the antibiotic solutions (antibiotic/CH_3_COO^−^ molar ratio = 2). The dispersions were left under mild stirring for 24 h at room temperature; then, they were centrifuged (12,000 rpm, 10 min). The precipitates obtained were washed three times with 30 mL of decarbonated water and the dry samples were analyzed by X-ray diffraction. The content of antibiotic was determined by thermogravimetric analysis and UV-Vis spectroscopy.

### 2.5. Synthesis of Ag/ZnAl-CFZ and Ag/ZnAl-NAL

The deposition of silver as AgCl NPs on the LDH exchanged with antibiotics was achieved with a two-step method that involves a contact with a chloride solution followed by the addition of AgNO_3_ solution [27]. Specifically, to promote the formation of AgCl, the ZnAl-CFZ and ZnAl-NAL compounds were washed quickly with 5 mL of 0.2 M NaCl solution, favoring the exchange of some intercalated anions with chloride ions, washed two times with 30 mL of decarbonated water, and dried at room temperature under vacuum. Subsequently, 100 mg of the recovered samples were suspended in 5 mL of decarbonated water and mixed with 1.3 mL of 0.05 M AgNO_3_ solution. The mixtures were stirred for 6 h in the dark, then centrifuged (12,000 rpm for 15 min). The precipitates obtained were washed with decarbonated water and dried at room temperature in the dark; the samples were labeled as Ag/ZnAl-CFZ and Ag/ZnAl-NAL. The same treatments were also performed on ZnAl-Ac in order to have a reference material (Ag/ZnAl-Ac). The content of the antibiotic was determined by thermogravimetric analysis and UV-Vis spectroscopy.

### 2.6. Techniques

X-ray diffraction spectra (XRD) of the powdered samples were recorded with a PANanalytical X’PERT PRO diffractometer equipped with an X’Celerator detector (PANalytical, Royston, United Kingdom), operating at 40 KV, 40 mA.

Thermogravimetric analyses (TGA) were obtained using a Netzsch STA 490 C TG-DTA thermal analyzer (Netzsch, Selb, Germany), operating at 10 °C/min heating rate and 30 mL/min air flow.

The content of zinc, aluminum, and silver was determined through Inductively Coupled Plasma-Optical Emission Spectrometers (ICP-OES) using a Varian Inc 710-ES series instrument (Varian 700-ES series, Santa Clara, CA, USA). To perform the analyses, the powdered samples were dissolved in concentrated HNO_3_ and appropriately diluted in deionized water.

To determine the Br^–^ anions content, 100 mg of the LDH was equilibrated in a Na_2_CO_3_ solution (20 mL, 1 M) for 12 h. The solution was then analyzed for the Br^–^ by ionic chromatography system DIONEX DX500 by an electric conductivity detector (DIONEX, Sunnyvale, CA, USA). The experimental analysis conditions were column S4SC, eluent 1.7 mM NaHCO_3_ and 1.8 mM Na_2_CO_3_ solution, flux 0.7 mL/s, and 12.5 mM H_2_SO_4_ solution as the suppressor for the ionic conductivity detector.

The morphologies of LDH and silver particles were investigated using a Philips 208 TEM electron transmission microscope (Philips, Eindhoven, The Netherlands). To prepare the samples, a drop of the dispersion was deposited on a copper grid covered with a Formvar polymer film, then evaporated in the air at room temperature. The size distribution of silver nanoparticles was obtained by measuring about 100 particles using the ImageJ software (National Institutes of Health, Bethesda, MD, USA).

UV-Vis absorption spectra were recorded at room temperature in the range of 190 nm to 700 nm by means of an Agilent Model 8453 dual-beam spectrophotometer (Agilent Technologies, Santa Clara, CA, USA) with a quartz cuvette having an optical path of 1 cm. To determine the amount of antibiotic loaded in the samples, powdered samples were dissolved in concentrated HNO_3_ and appropriately diluted in deionized water. The absorbance was measured at λ = 270 nm for cefazolin and λ = 260 nm for nalidixic acid, respectively

FT-IR spectra were recorded using an FT-IR Shimadzu IR-8000 spectrophotometer equipped with a total attenuated reflectance FT-IR spectrum acquisition device (Shimadzu, Europa GmbH, Duisburg, Germany). The spectral range collected was 400 to 4000 cm^−1^ with a spectral resolution of 4 cm^−1^ acquiring 200 scans.

### 2.7. Release Assay

The release profiles of the antibiotics were determined by placing 5 mg of samples (ZnAl-CFZ, Ag/ZnAl-CFZ, ZnAl-NAL, or Ag/ZnAl-NAL) in 1 mL of acceptor medium under mild agitation. The content of antibiotic, in mg/mL, of each sample submitted to the release test, determined by TGA and UV-Vis analyses (see result and discussion), was 2.25 mg/mL for ZnAl-CFZ, 1.44 mg/mL for Ag/ZnAl-CFZ, 1.91 mg/mL for ZnAl-NAL, and 1.35 mg/mL for Ag/ZnAl-NAL. At predetermined interval times, 0.5 mL of the dispersion were taken, filtered to remove the suspended particles, and diluted to 2.5 mL within the same medium. The media used are deionized water and phosphate buffer (pH 7). The tests took place over 24 h at 37 °C, and the number of intercalated species released was estimated by intensities of the characteristic UV-Vis absorption bands measured at λ = 270 nm for cefazolin and λ = 260 nm for nalidixic acid, respectively.

### 2.8. Microbial Strains and Growth Conditions

Three microbial strains were used to test the antimicrobial activity of the sample: *Staphylococcus aureus* (ATCC 29213), *Streptococcus pneumoniae* (ATCC 20566), and *Pseudomonas aeruginosa* (ATCC 15692). The bacterial cultures were maintained in Müller Hinton agar medium (MHA). One day before the test, a single colony was inoculated into Müller Hinton broth (MHB) and incubated for 24 h at 37 °C. The microbial cells were collected by centrifugation, washed, counted by spectrometric analysis (OD600), and suspended to the required concentration in the appropriate medium.

### 2.9. Minimum Inhibitory Concentration Assay

The minimum inhibitory concentration (MIC) of bacterial growth was determined by the micro-dilution method in accordance with international standards approved by the Clinical and Laboratory Standards Institute/National Committee for Clinical Laboratory Standards (CLSI/NCCLS).

Specifically, cefazolin (CFZ), AgNO_3_, nalidixic acid (NAL), Ag/ZnAl-Ac, ZnAl-CFZ, Ag/ZnAl-CFZ, ZnAl-NAL, and Ag/ZnAl-NAL were dispersed in MHB. Serial dilutions, ranging from 0.11 to 250 μg/mL, of each compound were prepared in 96-well U-bottomed plates. Simultaneously, a bacterial suspension from an overnight culture was prepared at a concentration of approximately 10^4^–10^5^ CFU/mL. Afterwards, 100 µL of the suspension was added to each well. The plates were then incubated at 37 °C for 24 h. Once the MIC was determined, the obtained values were normalized for the antibiotic or silver content by the following formula:

$$\mathrm{MIC}\;\left(\mathrm{normalized}\right)=\frac{\mathrm{MIC}\;\times \;\mathrm{wt}\%\;\left(a.s.\right)}{100}$$

where wt% (a.s.) is the weight percentage of the active species immobilized on LDH.

### 2.10. Assessment of the Synergy of Antimicrobial Activity of Antibiotics and Silver

The synergism of antibiotics and silver in the form of AgNO_3_ was evaluated by the microdilution checkerboard method according to the CLSI/NCCLS guidelines. The synergic behavior is expressed as fractional inhibitory concentration index (FICI). Assays were performed in a 96-well plate with scalar dilutions of cefazolin or nalidixic acid in the horizontal lines and AgNO_3_ in the vertical columns. Subsequently, 100 µL of *S. aureus*, *S. pneumoniae*, or *P. aeruginosa* (10^4^–10^5^ CFU/mL) bacterial suspensions were added to each well. The plate was then incubated for 24h at 37 °C. After the incubation, the microbial growth in each well was evaluated by spectrophotometric reading (600 nm). Each checkerboard produced different combinations of compounds that inhibited the growth of the bacteria. The FICI value was calculated as follows:

FICI = FIC_A_ + FIC_B_ = C_A_^comb^/MIC_A_^sol^ + C_B_^comb^/MIC_B_^sol^

where MIC_A_^sol^ and MIC_B_^sol^ are the MICs of compounds A (antibiotics) and B (AgNO_3_), respectively, and C_A_^comb^ and C_B_^comb^ are the concentrations of A and B when used in combination. An FICI value ≤ 0.5 is considered synergistic (SN), a value of FICI > 0.5 to 1 is partially synergistic (PS), equal to 1 is additive (AD), in the range of 1–2 is indifferent (ID), and ≥2 produces an antagonistic effect (AN).

## 3. Results and Discussion

### 3.1. Preparation and Characterization of Silver/Antibiotic Loaded LDH

**Figure 1:**
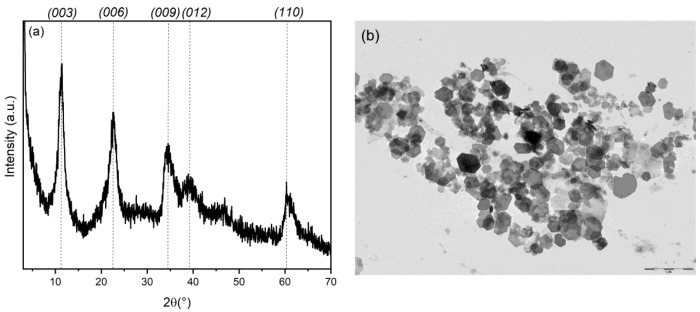
XRD pattern (**a**) and TEM image (**b**) of ZnAl-Br; the bar correspond to 1 μm.

Nanometric LDHs were prepared in reverse micelles according to the double microemulsion technique. Given the conditions of synthesis, in which the surfactant bromide counterions are diffused in the micellar water pool, the resulting LDH will be preferentially in bromide form (ZnAl-Br). These ions are easily exchangeable and provide a good precursor for the intercalation of other species [33]. The LDH obtained was investigated by X-ray diffraction and TEM analysis. Figure 1a shows the XRD profile of the sample. The (*003*) basal reflection is positioned at 11.26 (°) 2*θ*, corresponding to an interlayer distance of 7.9 Å, compatible with the presence of bromide ions. The low intensity and the amplitude of the diffraction peaks indicate the nanometric size of the particles. The nanometric dimensions hypothesized by X-ray diffraction are confirmed by TEM analysis (Figure 1b), in which fine crystals of hexagonal shape, with diameters ranging from 150 to 300 nm, can be distinguished.

**Figure 2:**
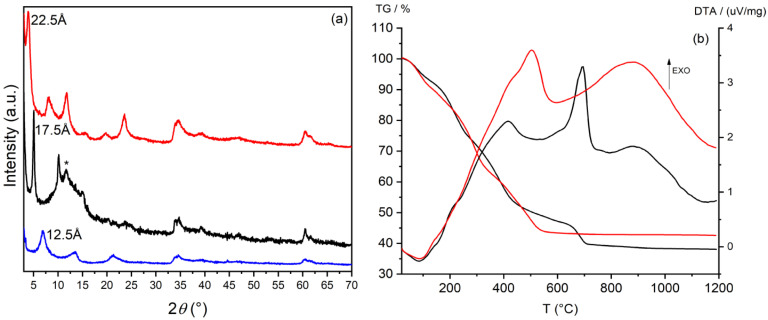
XRD patterns of ZnAl-Ac (blue line); ZnAl-CFZ (black line) (the asterisk indicates the presence of ZnAl-OH phase); ZnAl-NAL (red line) (**a**). TGA and DTA curves of ZnAl-CFZ (black line) and ZnAl-NAL (red line) (**b**); operative conditions: heating rate: 10 °C/min, air.

To promote the intercalation of larger species, the basal space of LDH was expanded by substituting the bromide ions with acetate ions through an ion exchange reaction. After the incorporation of acetate, the interlayer distance of ZnAl-Br increased from 7.9 Å to 12.5 Å (Figure 2a, blue pattern). The ZnAl-Ac sample obtained was used as a precursor for the incorporation of the selected antibiotics. The conditions of intercalation of each species were tailored according to their specific solubility, as reported in the experimental section. Figure 2a shows the diffraction patterns of ZnAl-CFZ and ZnAl-NAL compared to those of the pristine LDH in acetate form. In ZnAl-CFZ the interlayer distance is 17.5 Å (Figure 2a, black pattern), compatible with a bi-layered arrangement of the cefazolin molecules [36]. The peak at 7.57 Å is instead attributed to hydroxide groups, deriving from the basic solution in which the antibiotic is dissolved, that work as competing guest species against the CFZ anions. The intercalation of nalidixic acid leads to the formation of a compound with an interlayer distance 22.5 Å (Figure 2a, red pattern). The large *d*-space of this second compound can be again explained by suggesting a double layer distribution of the nalidixic ions within the interlayer space of LDH [37].

The thermal behavior of the samples was investigated by TGA and DTA (Figure 2b). The first weight loss at 120 °C is ascribable to the hydration water; the next two weight losses are overlapped and due to the dehydroxylation of the layer and to exothermic oxidation of the organic moieties. ZnAl-NAL reach a constant weight at 600 °C while ZnAl-CFZ undergoes a further weigh loss; the presence of sulfur in CFZ molecules provokes the formation of ZnSO_4_, which at about 650 °C decomposes into ZnO and SO_3_. The TGA was coupled with a UV-Vis measurement to determine the weight percentage of antibiotic loaded and the empirical formulae of the samples, reported in Table 1.

**Figure 3:**
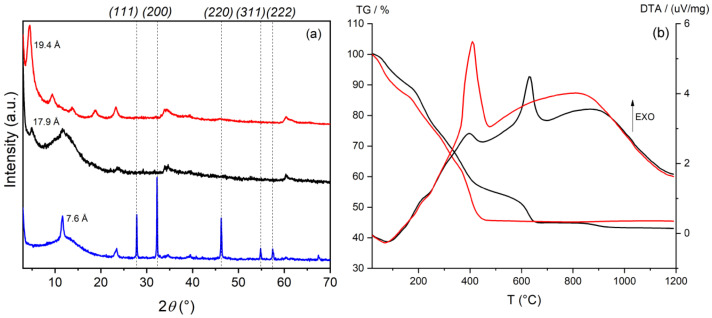
(**a**) XRD pattern of Ag/ZnAl-Ac (blue line), Ag/ZnAl-CFZ (black line), and Ag/ZnAl-NAL (red line). The crystalline phases reported belong to the cubic phase of AgCl NPs (PDF number: 85-1355). (**b**) TGA and DTA of Ag/ZnAl-CFZ (black line), Ag/ZnAl-NAL (red line), operative conditions: heating rate: 10 °C/min, air.

In order to verify the possible synergistic effect between each antibacterial agent and silver, AgCl NPs were deposited on the surface of the LDH support. The deposition of AgCl NPs was performed by a two-step method: first the samples were washed with an NaCl solution to exchange some molecular anions with chloride ions, which act as precipitating agents for silver in the form of AgCl; then, the samples were mixed with a AgNO_3_ solution [27]. The same procedure was applied to the ZnAl-Ac sample, obtaining a reference material. The diffraction pattern of the Ag/ZnAl-Ac composite is shown in Figure 3a. Reflections falling in the 25–60° 2*θ* range were assigned to the *(111)*, *(200)*, *(220)*, *(311)*, and *(222)* cubic phase of AgCl (ICDD Powder Diffraction File (PDF) number: 85-1355). The narrow amplitude of the peaks indicates the formation of fairly large crystalline domains and, therefore, of particles with dimensions in the order of a hundred of nanometers. The formation of silver NPs as a consequence of light irradiation cannot be excluded, even if no reflections are detected by XRD. Note that the treatment of the sample with silver nitrate provokes the exchange of acetate anions, weakly retained by LDH, with other anions in the solution, most likely carbonate or hydroxide anions due to the interlayer distance of the recover solid of 7.6 Å.

A different behavior was observed in the ZnAl-CFZ and ZnAl-NAL compounds after the treatment with NaCl and AgNO_3_ (Figure 3a); the addition of these reagents did not generate new diffraction peaks, indicating that the AgCl NPs or possible Ag NPs formed have very small crystalline domains.

The TGA and DTA of the Ag loaded samples (Figure 3b) are close to those of the pristine samples, the main differences being in the higher residual due to the presence of silver. TGA, ICP, and UV-Vis analyses were combined to obtain the composition of the composites in terms of weight percentage of antibiotic and silver, and the data are shown in Table 2.

Noteworthy is the yellow-orange color assumed by the Ag/ZnAl-CFZ composite. A solid with the same color and insoluble in aqueous solution was obtained by mixing a solution of CFZ with AgNO_3_, suggesting the presence of CFZ-Ag complexes. The formation of β-lactamase complexes with transition metals is already reported in literature; in particular, it is known that CFZ can act as a tetradentate ligand, complexing the metal through the carboxylate, amide carbonyl, tetrazole, and 1,3,4-thyodiazole groups. The coordination sphere of the complex can be completed by a chloride anion [38]. From these observations we can state that, upon the addition of AgNO_3_, some silver ions and some intercalated chloride anions react with the superficial cefazolin, taking part into the formation of the Ag-CFZ-Cl complex and imparting the typical yellow-orange coloration to the compound.

**Figure 4:**
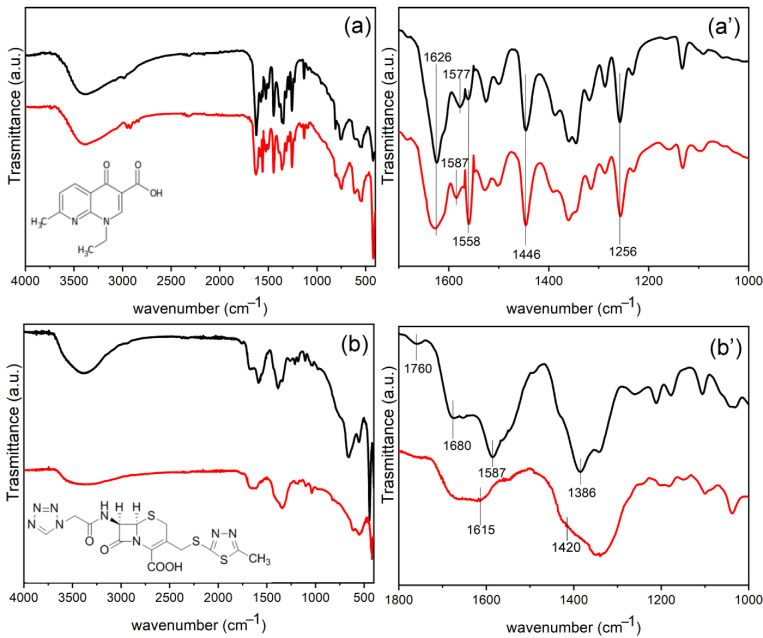
FT-IR of ZnAl-NAL (black line) and Ag/ZnAl-NAL (red line) (**a**), enlargement in the spectral range 1700–1000 cm^−1^ (**a’**); FT-IR of ZnAl-CFZ (black line) and Ag/ZnAl-CFZ (red line) (**b**), enlargement in the spectral range 1800–1000 cm^−1^ (**b’**). The structural formulae of nalidixic acid (**a**) and cefazolin (**b**) are reported.

To obtain more insight into possible interactions between silver and antibiotics, the FT-IR spectra of LDH intercalated with antibiotics and after silver loading were recorded and compared in Figure 4. In all the FT-IR spectra, the contribution of the LDH layers can be recognized. Specifically, the broad band centered at 3380 cm^−1^ is ascribable to the O-H stretching of the layers and the intercalated water molecules involved in hydrogen bonds; the bands positioned in the spectral range 400–700 cm^−1^ are generated from the Al–OH and Zn-OH translation modes.

FT-IR of ZnAl-NAL (Figure 4a,a’) shows bands below 3000 cm^−1^ assigned to the stretching of the aliphatic C-H of methyl and ethyl groups of NAL. The bands at 1576 and 1390 cm^−1^ are due to the asymmetric stretching vibrations of the C-O bonds of COO^−^ groups of the NAL anions, attesting the interaction of NAL with LDH with the carbolyxic groups. The strong band at 1626 cm^−1^ is due to C=O stretching of the pyridone. The bands at 1558 cm^−1^ and 1446 cm^−1^ are attributed to C-C stretching of the aromatic ring. In addition, the characteristic peak at 1256 cm^−1^ is attributed to the stretching vibration of the C–N group [39]. The addition of silver in Ag/ZnAl-NAL does not dramatically change the FT-IR spectrum; however the C=O band of the pyridone appears broader and the asymmetric stretching vibrations of COO^−^ groups are shifted to 1586 cm^−1^, suggesting that both C=O and COO^−^ groups are involved in different interactions such as with silver. From this evidence, the formation of NAL-Ag complexes cannot be excluded [40].

FT-IR spectrum of ZnAl-CFZ (Figure 4b,b’) shows the asymmetric and symmetric stretching of COO^−^ at 1587 and 1386 cm^−1^, respectively, attesting that here as well the interaction of CZF with LDH occurs via carboxylic groups. The peaks at 1680 and 1760 cm^−1^ are assigned to the stretching of C=O of the cyclic amide and β-lactam ring, respectively [38]. The presence of silver in Ag/ZnAl-CFZ causes significant modifications in the FT-IR spectrum. The COO^−^ asymmetric and symmetric stretching modes shift to 1615 cm^−1^ and 1420 cm^−1^, respectively. Moreover, these bands and the band of the C=O stretching of the amide become less intense and very broad. Finally, the band of C=O stretching of β-lactam ring is almost missing. All these observations suggest the formation of Ag-CFZ complexes, in which at least carboxylate and amide carbonyl are involved in interactions with silver that restrict the vibration modes of these groups [38].

**Figure 5:**
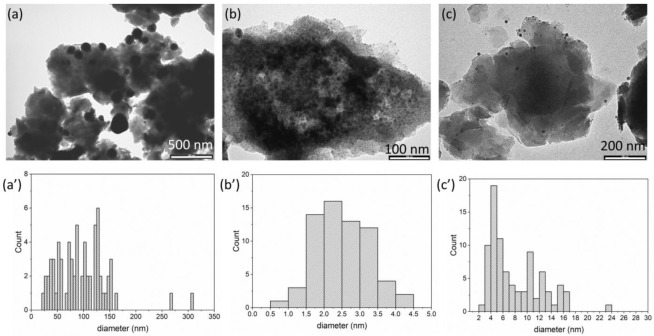
TEM images and size distribution profile of silver NPs of: Ag/ZnAl-Ac (**a**,**a’**); Ag/ZnAl-CFZ (**b**,**b’**); Ag/ZnAl-NAL (**c**,**c’**).

TEM images of the silver loaded composites and the size distribution are reported in Figure 5. The samples are very different from each other: Ag/ZnAl-Ac shows aggregates of LDH particles covered by silver chloride crystals having a wide size distribution centered at 96.3 nm (Table 2 and Figure 5a,a’); very small particles with an average size of 2.5 nm and with narrow size distribution are instead produced over the surface of Ag/ZnAl-CFZ (Table 2 and Figure 5b,b’); the TEM image of Ag/ZnAl-NAL (Table 2 and Figure 5c,c’) shows the formation of particles with an average size of 8.0 nm. For the last two samples, the darker particles can be traced back to silver species; however, the data collected do not allow us to distinguish between Ag NPs or AgCl NPs.

### 3.2. Release Profiles

**Figure 6:**
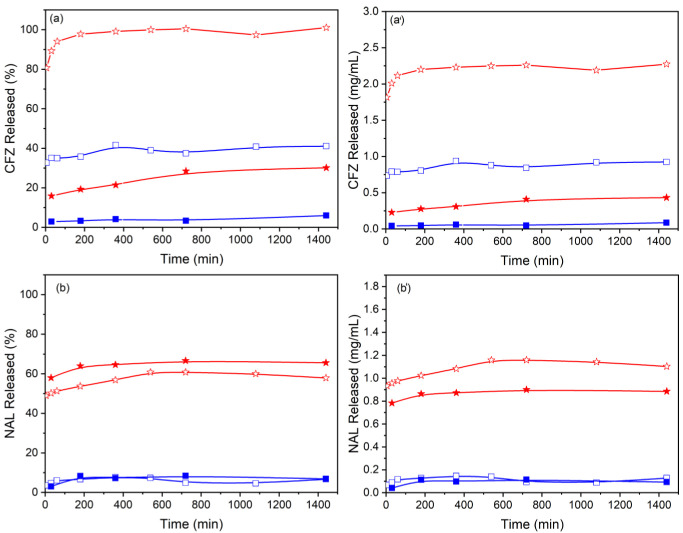
Release profiles of antibiotics in PBS (red) and water (blue) from ZnAl-CFZ (empty symbols) and from Ag/ZnAl-CFZ (full symbols) (**a**,**a’**) and from ZnAl-NAL (empty symbols) and Ag/ZnAl-NAL (full symbols) (**b**,**b’**).

The ability of LDH to release CFZ and NAL was studied in two different acceptor media: water and phosphate buffer (pH 7) at 37 °C. Figure 6 shows the release profiles of samples with and without Ag NPs, plotted both as percentage and as mg of antibiotic released as a function of time. In all the cases, the curves reach a plateau after 3 h, maintaining a stable value throughout the time of investigation (24 h). The maximum percentage of drug released is higher in phosphate buffer than water; in particular, the release is complete in PBS for sample ZnAl-CFZ and reaches 41% in water. These values appear extremely reduced for Ag/ZnAl-CFZ, where the equilibrium rate of the released species remains around 30% in PBS and drops to 5% in water. The weight percentage of silver released after 24 h was also evaluated and resulted 1% and 1.8% of the total in water and PBS, respectively. The differences in the release profiles and the low amount of released silver could be related to the low solubility of Ag-CFZ-Cl complexes that are formed in the sample, as evidenced by the color taken by the solid and by the FT-IR spectra. Similar trends can be described for nalidixic acid. This time, the release in PBS is not complete due to the low solubility of nalidixic acid in aqueous solutions (0.328 mg/mL at pH = 7) [41]. In PBS at pH 7, a fraction of the released NAL anions is converted into nalidixic acid (pKa = 6) [42] which, due to the low solubility, precipitates forming a saturated solution. The release curves of Figure 6b show the percentage of NAL released, the maximum release from ZnAl-NAL is 61% and from Ag/ZnAl-NAL is 67% of the total NAL content corresponding to 1.2 mg/mL and 0.9 mg/mL, respectively. Therefore, slight discrepancies can be observed in the release profiles after silver deposition on the particle surfaces. The lower release of NAL from Ag/ZnAl-NAL (more evident in Figure 6b’) could be due to the involvement of a fraction of NAL in the formation of less soluble Ag-NAL complexes. The weight percentage of silver released after 24 h was 32.5% and 1% of the total in water and PBS, respectively. The low amount of silver in PBS could be ascribed to re-precipitation of silver cations as silver phosphate. To explain the effect of different media in the release rate of the same compound, a few considerations have to be made; the release process in LDH is associated with ion exchange reactions involving the hosted anions and the ones diffused in the medium. As a consequence, the release kinetics are influenced by several factors including drug-LDH interactions, LDH affinity with the anions present in the medium, and the size of the LDH particles [43]. On the basis of these observations, the reduced release in water can be justified assuming that antibiotics are released due to the ion exchange with some carbonate anions resulting from the dissolution of atmospheric CO_2_ in water. In the case of PBS, significantly higher releases can be explained by the strong affinity of LDH for hydrogen phosphate and dihydrogen phosphate anions that are present in higher concentration with respect to the carbonate ions in the water medium. Moreover, the burst effect observed during the first hour is due to the release of the antibiotics present on the LDH surface and on the edges of the interlayer region; later, the internal anions are also released [43].

### 3.3. Antimicrobial Tests

In a first series of experiments, the minimum inhibitory concentration was evaluated for plain CFZ, NAL, AgNO_3_, and Ag/ZnAl-Ac. Another set of tests was executed over LDH intercalated with individual antibiotics (ZnAl-CFZ and ZnAl-NAL) and over the same samples functionalized with silver (Ag/ZnAl-CFZ and Ag/ZnAl-NAL). The results, reported in Table 3 and in Figure S1 in the Supplementary Materials, indicate that AgNO_3_ is active against all the examined bacteria. This activity is strongly reduced when silver is deposited on LDH in the form of AgCl (Ag/ZnAl-Ac); its availability, in fact, is dependent on the solubility of AgCl (about 10^−5^ mol/L in water) and on the dimension of the AgCl NPs (about 100 nm).

Regarding the antibiotic activity, plain CFZ results active towards gram-positive bacteria, especially *S. aureus* (6.2 µg/mL), and, as expected, has no influence towards gram-negative *P. aeruginosa* (1000 µg/mL), while the MIC values of NAL are noteworthy against *S. pneumoniae* (3.3 µg/mL). After the intercalation in LDH, the inhibitory effect of both compounds is reduced, with the exception of that of ZnAl-CFZ against *S. aureus* (1.3 µg/mL). To investigate the activity of the Ag/ZnAl-CFZ and Ag/ZnAl-NAL composites, the results were normalized with respect to the drug (CFZ or NAL) and to the silver contents. In the first case, the MIC of Ag/ZnAl-CFZ partially rises if compared to that of pure CFZ against gram-positives but becomes significantly lower against the gram-negative (40.4 µg/mL), approaching the AgNO_3_ value. The activity of the same composite normalized with respect to silver is comparable to that of AgNO_3_ for *S. aureus* (8.0 µg/mL) and *S pneumoniae* (10.7 µg/mL) and considerably reduced against *P. aeruginosa* (12.1 µg/mL), suggesting a synergistic effect between the two antibacterial agents. The strong discrepancy of the silver activity in the precursor (Ag/ZnAl-Ac) and in the samples containing the antibiotics (Ag/ZnAl-CFZ and Ag/ZnAl-NAL) can be explained in the view of the different nature of silver in the two composites. As previously discussed, in Ag/ZnAl-Ac, the majority of silver is in the form of AgCl particles of about 100 nm, while, in Ag/ZnAl-CFZ, part of the silver is complexed with CFZ and another part is present as Ag NPs of very small dimension (2.5 nm), as shown by TEM images. Furthermore, the fraction of silver and cefazolin involved in the formation of the Ag-CFZ complex may not be available to exert antibacterial activity given the low solubility of the complex in water [38]; however, the very small dimension of the Ag NPs partially compensate for it, offering a large surface area of interaction.

The activity of the composite Ag/ZnAl-NAL, normalized for NAL content, is higher than that observed for plain NAL and especially than that obtained for ZnAl-NAL against all the strains examined. These values improve even more when normalized with respect to the silver for *S. pneumoniae* (in which the MIC value is reduced to 0.9 µg/mL from 10.8 µg/mL) and *P. aeruginosa* (in which the MIC value is reduced to 21.4 µg/mL from 31.6 µg/mL. These data are even more remarkable if compared to that of the reference sample, Ag/ZnAl-Ac. The activity of ZnAl-LDH was investigated in a previous work and no antibacterial activity was shown [27]. Again, the improvement in the antibacterial activity can be related to the different dimensions of the silver species in the two composites; however, other aspects to evaluate are the nature of the antibiotics, the antimicrobial mechanism involved in the bacterial inhibition, and the target bacteria. The two drugs examined are part of the β-lactam (cefazolin) and quinolone (nalidixic acid) family. The first attacks the cell, binding covalently to the enzymes responsible for building the bacterial cell wall [44]. On the other hand, the mode of action of quinolones is not confined to the surface of the cell; quinolones act on the inside, inhibiting the DNA gyrase activity and preventing the supercoiling of DNA necessary for compacting chromosomes [45]. In regards to silver, its action can be considered ubiquitarian; in fact, the antibacterial mechanism of silver is divided into contact killing and ion-mediated killing [46]. Ag NPs are electrostatically attracted by negatively charged cell membranes and, once anchored to the cell wall, destabilize and infiltrate them, promoting the leakage of the cellular content. At the same time, silver can penetrate into the cell in the form of Ag^+^_,_ threatening multiple targets [47]. For those antibiotics that act intracellularly, such as quinolones, silver can be imagined as a weakening agent that undermines the microbe defense wall system and promotes the access of the antibiotic within the cell, increasing its effectiveness. By contrast, when the antibiotic action is extracellular, its efficiency is less influenced by changes in the cell permeability, and the antimicrobial action is mainly assigned to silver [48]. This could explain the rise in the MIC value for Ag/ZnAl-CFZ sample against *S. aureus* and *S. pneumonia* when compared to plain cefazolin and the significant enhancement of the antimicrobic activity when silver is coupled with nalidixic acid. Other observations should regard the nature of the target bacteria and the mechanisms employed by the microorganism to develop resistance; however, despite all these specific evaluations, this study demonstrates that silver has a potential synergistic effect when placed in combination with other species to target a broad spectrum of both gram-positive and gram-negative bacteria.

In comparison, the combined effect of plain CFZ or NAL and AgNO_3_ was studied in checkerboard experiments, as reported in Table 4. No synergistic effect between antibiotic and silver ions was observed even if a partially synergistic (PS) effect is shown by AgNO_3_/NAL on *S. aureus* and by AgNO_3_/CFZ on *P. aeruginosa*. However, the effect of silver combined with CFZ or NAL on the last bacterium is enhanced when the two antimicrobials are immobilized on the LDH. Indeed, the positive charge of the LDH surface is able to attract negatively charged bacterial cells by promoting bacterial adhesion on LDH, and the close contact between the antimicrobial agents and the bacteria supports the action of the active species [49].

## 4. Conclusions

Nanometric LDHs represent interesting systems that combine the reactivity of micrometric LDHs with the nanometric size (smaller than that of cells), making them excellent materials for use in nanomedicine. With the aim of preparing a nanometric system capable of carrying a combination of antibiotics and silver, nanometric ZnAl-LDH was functionalized in a two-step process. In the first step CFZ or NAL antibiotics in anionic form were intercalated via anionic exchange in LDH; in the second step, the growth of silver nanoparticles was induced by the presence of chloride ions on the LDH surface. The formation of silver NPs, which can be traced back to metallic silver or AgCl, was confirmed by TEM, which showed the presence of NPs smaller than 10 nm. The release profiles of the intercalated antibiotics were investigated in water and in PBS. The presence of phosphate anions in PBS favored increased antibiotic release of both CFZ and NAL. The presence of silver in Ag/ZnAl-CFZ lowered CFZ release in all media due to the formation of insoluble CFZ-Ag complexes on the solid surface. The activity of both the LDH-supported antibiotics, in combination with silver (sample Ag/ZnAl-CFZ and Ag/ZnAl-NAL), was markedly improved against *P. aeruginosa* (gram-negative), while lower MIC values were obtained with Ag/ZnAl-NAL against *S. aureus* and *S. pneumoniae* (gram-positives). Ag NPs appear to assist the antibacterial activity of antibiotics most likely promoting the access of antibiotics inside bacterial cells. More interestingly, the antibiotic activity of Ag/ZnAl-CFZ and Ag/ZnAl-NAL is higher than that of antibiotics combined with silver in solution, studied in checkerboard experiments. LDH possibly promoted bacterial adhesion on its surface enhancing the action of active species.

## Figures and Tables

**Table 1:** Sample acronym, composition, and weight percentage of antibiotic (A) loaded.

Sample	A	Composition [Zn_0__.__72_Al_0__.__28_(OH)_2_](A)_x_(OH)_y_·nH_2_O			A (wt%)
		x	y	n	
ZnAl-CFZ	CFZ	0.19	0.09	0.8	45.0
ZnAl-NAL	NAL	0.28	0	0.9	38.1

**Table 2:** Weight percentage of intercalated antibiotics and silver deposited over each sample; the diameter of silver NPs (in form of Ag or AgCl) determined by TEM is also reported.

Sample	Antibiotic (wt%)	Ag (wt%)	Ag/AgCl NPs Diameter (nm)
Ag/ZnAl-Ac	-	8.2	96.3 ± 51.0
Ag/ZnAl-CFZ	28.7	8.6	2.5 ± 0.7
Ag/ZnAl-NAL	27.0	10.3	8.0 ± 4.3

**Table 3:** MIC values of the samples against *S.* *aureus*, *S. pneumoniae*, and *P. aeruginosa*.

Sample	MIC(µg/mL)					
	*S. aureus*		*S. pneumoniae*		*P. aeruginosa*	
AgNO_3_	10.8		10.8		31.6	
CFZ	6.2		26.0		1000.0	
NAL	83.3		3.3		250.0	
Ag/ZnAl-Ac	375.0		1000.0		666.7	
ZnAl-CFZ	1.3		333.3		1000.0	
Ag/ZnAl-CFZ	26.9 ^(a)^	8.0 ^(b)^	35.9 ^(a)^	10.7 ^(b)^	40.4 ^(a)^	12.1 ^(b)^
ZnAl-NAL	190.3		7.93		253.7	
Ag/ZnAl-NAL	67.5 ^(a)^	25.6 ^(b)^	2.3 ^(a)^	0.9 ^(b)^	56.3 ^(a)^	21.4 ^(b)^

^(a)^ values normalized for the antibiotic content; ^(b)^ values normalized for the silver content.

**Table 4:** Fractional Inhibitory concentration Index of AgNO_3_ and antibiotics. SN—synergistic (≤0.5); PS—partially synergistic (>0.5 to 1); AD—additive (equal to 1); ID—indifferent (>1 to <2); AN—antagonistic (≥2) on the basis of FICI.

Antibacterial Combination	FICI (mg/mL)	
	AgNO_3_/NAL	AgNO_3_/CFZ
*S. pneumoniae*	1.4 (ID)	1.2 (ID)
*S. aureus*	0.9 (PS)	1 (AD)
*P. aeruginosa*	1.4 (ID)	0.75 (PS)

## Data Availability

Not applicable.

## Supplementary Materials

The following supporting information can be downloaded at: https://www.mdpi.com/article/10.3390/ma15030932/s1, Figure S1: MIC values of the samples against *S.* *aureus*, *S. pneumoniae* and *P. aeruginosa*.
