# Supplementary material for: Overcoming Antibiotic Resistance: Playing the ‘Silver Nanobullet’ Card
Source: Materials (Basel). 2022 Jan 26;15(3):932. doi: 10.3390/ma15030932 (PMC8839980; doi:10.3390/ma15030932)
Supplement: Supplementary file 1 [file materials-15-00932-s001.zip › materials-1551063-supplementary.pdf]

materials

MDPI

Supporting Information

Overcoming Antibiotic Resistance: Playing the 'Silver Nanobullet' Card

Morena Nocchetti [1,*], Elisa Boccalon [2], Monica Pica [1], Nicoletta Maria Rosaria Giordano [1], Francesco Finori [3], Donatella Pietrella [4] and Antonio Cipiciani [3]

[1] Department of Pharmaceutical Sciences, University of Perugia, Via del Liceo, 1, 06123 Perugia, Italy; monica.pica@unipg.it (M.P.); nicoletta.giordano@hotmail.it (N.M.R.G.)
[2] Department of Industrial Engineering, University of Salerno, Via Giovanni Paolo II, 132, 84084 Fisciano, Salerno, Italy; elisa.boccalon@gmail.com
[3] Department of Chemistry, Biology and Biotechnology, University of Perugia, Via Elce di Sotto, 8, 06123 Perugia, Italy; cescofinori@hotmail.it (F.F.); antonio.cipiciani@libero.it (A.C.)
[4] Microbiology and Clinical Microbiology, Department of Medicine and Surgery, University of Perugia, Piazzale Gambuli, 1, 06129 Perugia, Italy; donatella.pietrella@unipg.it
* Correspondence: morena.nocchetti@unipg.it

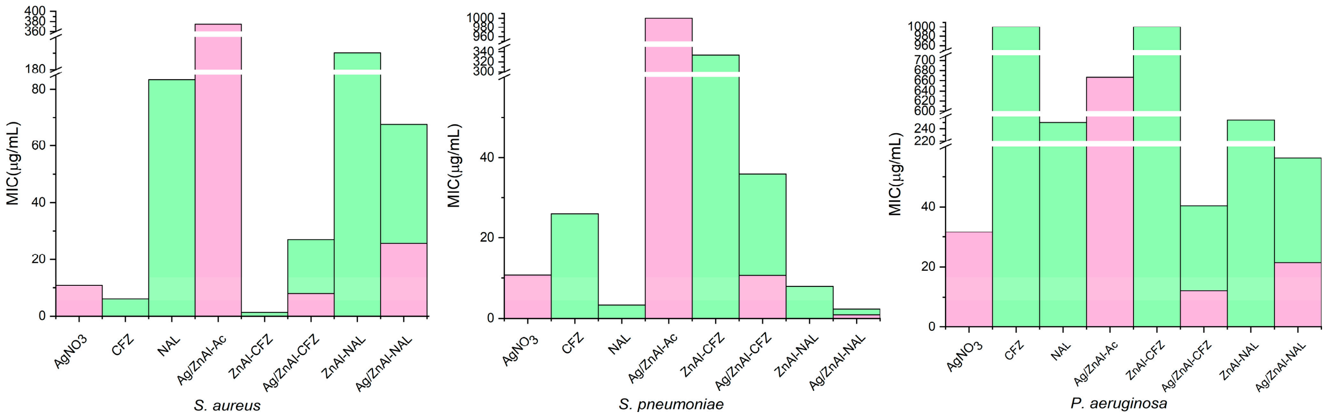

Figure S1. MIC values of the samples against *S. aureus*, *S. pneumoniae* and *P. aeruginosa*. Values normalised for the antibiotic content (green columns), values normalised for the silver content (pink columns).
